# Large‐scale reproductive loss in sheep due to Border disease virus infection, New South Wales, Australia

**DOI:** 10.1111/avj.70037

**Published:** 2025-11-21

**Authors:** K Parrish, ZB Spiers, MS Hazelton, KH Walker, E Duggan, W Graham, DS Finlaison, PD Kirkland

**Affiliations:** ^1^ NSW Department of Primary Industries Elizabeth Macarthur Agriculture Institute Woodbridge Road Menangle New South Wales 2568 Australia; ^2^ Riverina Local Land Services 93 Main Street Young New South Wales 2594 Australia; ^3^ Riverview Coolac New South Wales 2727 Australia

**Keywords:** border disease virus, congenital tremors, hairy lambs, pestivirus, reproductive loss, sheep, stillbirth

## Abstract

Border disease viruses (BDV) and bovine viral diarrhoea viruses (BVDV) are members of the Pestivirus genus in the family *Flaviviridae*. While BVDV is one of the most significant endemic viral infections of cattle in Australia, BDV infection is generally considered to be uncommon in Australian sheep. This study describes the widespread detection of BDV on two properties in southern New South Wales following an investigation into poor pregnancy rates, resorbing foetuses and stillborn lambs. Extensive cross‐sectional serological studies identified a high seroprevalence in some groups of sheep and low prevalence in others, demonstrating both the extent of infection and the number of susceptible breeding sheep remaining at risk. BDV‐specific qRT‐PCR confirmed BDV infection of stillborn lambs, and a large number of ‘hairy’ lambs were confirmed as BDV infected by use of a pestivirus antigen ELISA at marking. In a group of BDV persistently infected lambs that were monitored for 12 months, postweaning survival was low, with 21 of 120 still alive at 5 months of age and 14 still alive at 12 months of age. This study highlights the potential impact BDV can have on production and how management strategies, including breeding young primiparous ewes under intensive management conditions can result in large‐scale virus transmission and disease.

**Abbreviations**
AGID: agar gel immunodiffusion test
BCS: body condition score
BDV: border disease virus
BVDV: bovine viral diarrhoea virus
CRL: crown‐rump length
ELISA: enzyme‐linked immunosorbent assay
NSW: New South Wales
PACE: pestivirus antigen capture ELISA
PI: persistently infected
qRT‐PCR: real‐time reverse transcription polymerase chain reaction
VN: virus neutralising
VNT: virus neutralisation test

Viruses belonging to the Border disease virus (BDV) species (now known taxonomically as *Pestivirus ovis*) are RNA viruses within the *Pestivirus* genus in the family *Flaviviridae*.^1^ Differing strains of BDV are found in sheep populations around the world.^2^ These viruses are predominantly pathogens of sheep but may occasionally infect other small ruminants, pigs and cattle.^3^, ^4^, ^5^, ^6^ Like the related bovine viral diarrhoea viruses (BVDV) in the taxonomic species *P. bovis*, *P. tauri* and *P. braziliense*, they have the most significant clinical impact by causing reproductive disease following infection of the foetus at critical stages of pregnancy.^7^, ^8^

Transmission of BDV to nonpregnant animals usually results in a subclinical infection with occasional isolates shown to produce pyrexia, leukopaenia and elevated mortality.^3^, ^9^, ^10^, ^11^ In contrast, infection of pregnant ewes with BDV can cause substantial reproductive losses as a consequence of transplacental infection. The immunological capacity of the foetus, which can typically respond to an antigenic stimulus commencing between 60 and 85 days of its 150‐day gestation, influences the outcome of infection.^12^ Infection before immunocompetence can result in foetal death, which becomes apparent as reduced conception rates, resorption, abortion and stillbirths. In lambs that are infected before immunocompetence is established, the virus is widespread in most organs and surviving lambs are persistently infected (PI) for life. The clinical presentation of these PI lambs can be variable but is typically characterised by a hairy fleece, weakness, tremors or ataxia and abnormal body conformation, resulting in the terms ‘hairy’ or “hairy shaker” lambs.^3^ The variability in the presentation of PI lambs can be influenced by the breed of sheep, the virulence and strain of virus and the time at which infection was introduced into the flock.^3^, ^7^, ^13^, ^14^ While many PI lambs die soon after birth, significant mortality rates are also observed after weaning, although there are reports of survival of PI lambs up to 5.5 years.^12^ These lambs are a continual source of infectious virus for other sheep, shedding virus in bodily secretions. Their identification is critical in any control programme.^12^ Infection after immunocompetence, in late gestation, typically results in apparently normal lambs that are free of BDV but with antibodies against this virus. However, some of these non‐PI lambs can be stillborn, weak or die in early life.^3^, ^14^

Flock management plays an important role in the occurrence of disease due to BDV because of the potential to either segregate or mix animals of susceptible or PI status, thereby influencing virus transmission patterns. In Australia, sheep are generally managed in extensive pasture‐based systems with variations in natural feed availability due to temperature and rainfall patterns, with feed supplementation as needed.^15^ Confined, intensively managed feeding areas (also referred to as “drought lots”) are a valuable management strategy in drought, to provide full feeding and resting of pastures. However, these management practices generate close animal contact and result in more efficient virus transmission.^16^ Additionally, there has been a trend in the Australian sheep industry over the last 10–15 years towards joining primiparous (maiden) lambs at approximately 7–9 months of age to increase lifetime reproductive performance and farm profitability.^17^, ^18^, ^19^ Improving the reproductive performance of maiden ewes has been identified as a priority for the Australian sheep industry, but there is considerable variation in reproductive outcomes reported, resulting in significant emphasis on joining management to maximise outcomes.^20^, ^21^, ^22^, ^23^ In contrast to the well‐studied impacts of nutrition, liveweight and time of joining, there are few studies on the impact of endemic diseases on the reproductive outcomes of maiden ewes.^21^ The joining of maiden ewes at a young age limits the potential window for them to acquire natural immunity to a range of pathogens before pregnancy.^24^ Despite receiving colostrum containing antibodies to infectious agents circulating in a flock, as this passive immunity will have usually waned before joining, there will be a very short time interval in which they can acquire immunity naturally before becoming pregnant.

During investigations of early embryonic loss and abortions on two large sheep‐raising properties in the South‐Western Slopes region of New South Wales (NSW), BDV was detected. In the 12 months before these investigations, this region experienced drought, requiring the provision of supplementary feed, often under intensively managed conditions in confinement feeding areas (drought lots). Both properties ran commercial Composite ewe flocks, a crossbred sheep that is primarily a meat‐producing animal with increased mature size, high fecundity and enhanced lamb survival.^25^, ^26^ This study describes the subsequent investigation of BDV in association with reproductive losses initially in maiden Composite ewes, but then more extensively across the entire sheep populations on each farm.

## Materials and methods

### History—Property 1

This property ran stud Merino and commercial Composite breeding operations, with more than 20,000 ewes joined annually. Animal introductions included wether teasers from another property under the same ownership and rams from two other sources. Historically, a small number of small and hairy lambs had been observed on the properties under investigation. All ewes were vaccinated against Campylobacter spp. (Ovilis Campyvax [Coopers©]) before joining. In mid‐March 2019, maiden Composite ewes were joined at 7–8 months of age, approximately 3–4 weeks after the joining of older ewes. Rams were joined at a rate of 4% and removed after a six‐week joining period. Ewes were joined on native pastures with the provision of supplementary feed. After joining, ewes were provided full feeding in a confinement feeding area to maintain a body condition score of 2.5–3.2 until ultrasound scanning for pregnancy in late June 2019. Scanning results for a single group of 3220 maiden Composite ewes showed an initial pregnancy rate of 72% with approximately 3% of pregnancies undergoing resorption. Two weeks later, a follow‐up ultrasound scan of approximately 10% of the previously identified pregnant ewes identified an additional 4.5% with resorbing foetuses.

### Specimen collection—Property 1

#### Foetal specimens

Due to the increasing proportion of foetal losses, two maiden Composite ewes (2018 born) with apparently dead foetuses were euthanased on 11 July 2019 and the uteri and contents were sent to the laboratory. The foetuses had a crown‐rump length (CRL) of 12 cm and 11.5 cm (6–8 weeks gestation^27^). A second submission of an aborted foetus from the same management group was submitted in early August 2019. Although severely autolysed, this foetus had a CRL of 21 cm (9–11 weeks gestation^27^). During lambing of the maiden Composite ewes, 10 full‐term stillborn lambs of varying condition were collected between late August 2019 (n = 7) and early September 2019 (n = 3). Table 1 summarises the 10 full‐term dead lambs submitted, and the tissues selected for testing.

**Table 1:** Diagnostic pestivirus results for 10 dead lambs from Property 1

Lamb:	1	2	3	4	5	6	7	8	9	10
Classification	Perinatal death	Perinatal death	Perinatal death	Perinatal death	Perinatal death	Perinatal death	Stillborn	Perinatal death	Perinatal death	Stillborn
Crown to rump length (cm)	39	41	36	37	41	42	60	42	45	49
Estimated gestation (weeks)	15–17	18–20	15–17	15–17	18–20	18–20	18–20	18–20	18–20	18–20
Hairy	No	No	No	No	Yes	No	No	Yes	Yes	No
Pestivirus qRT‐PCR^a^										
Lung	Negative	Negative	Negative	Negative	Negative	36.1	Negative	37.3	Negative	Negative
Spleen	36.5	Negative	34.0	Negative	Negative	Negative	Negative	Negative	Negative	Negative
Brain	Negative	33.9	Negative	Negative	34.9	Negative	Negative	35.0	38.2	Negative
Pericardial fluid	33.4	34.2	31.2	Negative	36.2	35.2	Negative	Negative	Negative	Negative
Thoracic fluid	34.6	37.6	32.4	Negative	Negative	35.7	Negative	Negative	Negative	Negative
BDV qRT‐PCR^a^										
Lung	28.3	30.4	29.7	Negative	29.4	27.7	Negative	29.6	29.9	Negative
Spleen	26.3	29.5	25.5	Negative	29.9	29.4	Negative	30.6	31.3	Negative
Brain	28.3	26.5	26.9	Negative	28.9	29.0	Negative	29.2	30.3	Negative
Pericardial fluid	23.9	22.6	20.3	Negative	25.6	25.0	Negative	36.1	26.8	Negative
Thoracic fluid	22.3	24.2	21.8	Negative	26.9	24.5	Negative	27.6	34.9	Negative
PACE^b^										
Lung	1.74	1.84	1.22	Negative	Negative	2.25	Negative	1.93	1.88	Negative
Spleen	1.67	1.90	1.02	Negative	1.26	2.16	Negative	1.87	2.25	Negative
Pericardial fluid	1.54	2.06	1.06	Negative	Negative	1.58	Negative	1.27	1.57	Inconclusive
Thoracic fluid	1.18	1.22	1.29	Inconclusive	Negative	1.84	Negative	1.18	1.76	Inconclusive
AGID^c^										
Pericardial fluid	Negative	Negative	Negative	Negative	>3	Negative	Negative	Negative	Negative	Negative

^a^ Real‐time reverse transcription polymerase chain reaction (qRT‐PCR): Cycle threshold (Ct) values <40 are considered to be positive.

^b^ Pestivirus antigen capture ELISA (PACE): Corrected Optical Density (OD) values ≥0.4 are positive.

^c^ Agar gel immunodiffusion assay (AGID): Positive results are graded 1 to >3 depending on strength of reactivity relative to positive control.

These lambs were delivered in August–September 2019 by maiden Composite ewes that were born in 2018.

#### Flock seroprevalence

In late July 2019, 30 clotted blood samples were collected from maiden (born in 2018) Composite ewes identified by ultrasound scanning to have resorbing foetuses (n = 10), or not pregnant (n = 10) or pregnant (n = 10) (Table 2). Subsequently, between August and December 2019 samples were collected across the property for an extensive serological survey for antibodies to BDV, taking into account various ages (born in 2014–2019), breeds (Composite, Merino and Poll Dorset) and sex (ewes, rams and wether teasers) (Table 3).

**Table 2:** Pestivirus serology results of maiden Composite ewes (2018)—Property 1.

Cohort	AGID^a^	BDV VNT^b^	BVDV VNT^b^
Affected 1	Negative	<4	<4
Affected 2	3	16,384	512
Affected 3	>3	8192	256
Affected 4	3	4096	64
Affected 5	3	8192	64
Affected 6	3	8192	64
Affected 7	2	4096	128
Affected 8	3	4096	512
Affected 9	3	512	8
Affected 10	3	4096	32
Empty 1	>3	8192	Not tested
Empty 2	Negative	<4	Not tested
Empty 3	3	8192	Not tested
Empty 4	3	4096	Not tested
Empty 5	1	2048	Not tested
Empty 6	2	4096	Not tested
Empty 7	1	512	Not tested
Empty 8	>3	8192	Not tested
Empty 9	2	2048	Not tested
Empty 10	3	1024	Not tested
Pregnant 1	3	4096	Not tested
Pregnant 2	2	8192	Not tested
Pregnant 3	3	4096	Not tested
Pregnant 4	2	2048	Not tested
Pregnant 5	2	1024	Not tested
Pregnant 6	3	8192	Not tested
Pregnant 7	2	4096	Not tested
Pregnant 8	3	2048	Not tested
Pregnant 9	>3	8192	Not tested
Pregnant 10	3	16,384	Not tested

^a^ Agar gel immunodiffusion assay (AGID): Positive results are graded 1 to >3 depending on the strength of reactivity relative to the positive control.

^b^ Virus neutralisation test (VNT): Titres <4 are considered to be negative.

Serum samples from ewes identified as having resorbing foetuses (Affected), nonpregnant ewes (Empty) and unaffected pregnant ewes (Pregnant) were tested in the pestivirus agar gel immunodiffusion (AGID) and virus neutralisation (VN) tests for Border disease virus (BDV) and bovine viral diarrhoea virus (BVDV).

**Table 3:** Flock seroprevalence Property 1.

Cohort	Breed	Population	AGID^a^						
			Prevalence % (95% CI)	Negative	1	2	3	>3	Total
2014	Composite ewes	2165	80 (58–92)	4	5	11			20
2015	Composite ewes	3132	80 (58–92)	4	1	9	6		20
2016	Composite ewes	2307	75 (53–89)	5	3	6	6		20
2017	Composite ewes	3216	100 (84–100)	0	2	10	8		20
2018	Composite ewes	3220	93 (79–98)	2	2	7	15	4	30
Total		**14,040**	**86 (79–92)**	**15**	**13**	**43**	**35**	**4**	**110**
Mixed	Dorset rams	NA^b^	93 (70–99)	1	2	6	6		15
2017	Dorset rams	163	71 (57–82)	14		23	10	1	48
2018	Dorset rams	NA	89 (79–94)	8	2	35	25		70
2019	Dorset weaner rams (6 months old)	NA	0 (0–16)	20					20
Total		**NA**	**72 (64–78)**	**43**	**4**	**64**	**41**	**1**	**153**
Mixed	Composite rams	NA	47 (25–70)	8	2	5			15
2017	Composite rams	154	21 (10–37)	27		4	2	1	34
2018	Composite rams	NA	63 (53–72)	37	2	31	30		100
2019	Composite weaner rams (6 months old)	NA	0 (0–16)	20					20
Total		**NA**	**46 (38–53)**	**92**	**4**	**40**	**32**	**1**	**169**
2017	Wether teasers	98	13 (4–38)	13	1		1		15
Total		**98**	**13 (4–38)**	**13**	**1**		**1**		**15**
2014	Merino ewes	1140	15 (5–36)	17	1	2			20
2015	Merino ewes	1343	5 (1–24)	19		1			20
2016	Merino ewes	1488	0 (0–16)	20					20
2017	Merino ewes	1680	0 (0–16)	20					20
Total		**5651**	**5 (2–12)**	**76**	**1**	**3**	**0**	**0**	**80**
Mixed	Merino rams	87	10 (2–40)	9	1				10
Total		**87**	**10 (2–40)**	**9**	**1**				**10**

^a^ Agar gel immunodiffusion assay (AGID): Positive results are graded 1 to >3 depending on the strength of reactivity relative to the positive control.

^b^ NA: not available.

Pestivirus seroprevalence determined by agar gel immunodiffusion (AGID) test for both older (2014–2017) and maiden (2018) Composite ewes, ram groups, wether teasers and older (2014–2017) Merino ewes.

#### Identification of PI lambs from mature composite ewes

In late August 2019, the progeny of older (2014, 2015 and 2017) Composite ewes that had lambed 3–4 weeks before the maiden ewes were examined. Small and/or hairy lambs with goat‐like hair were identified and clotted blood, EDTA‐treated blood and tonsillar swabs were collected at lamb marking when lambs were approximately 2–8 weeks of age (Table 4).

**Table 4:** Pestivirus results for different sample types from persistently infected lambs from mature Composite ewes at marking at Property 1

Lamb	Appearance	EDTA blood		Serum				Tonsillar swab	
		Pestivirus PCR^a^	BDV PCR^a^	Pestivirus PCR^a^	BDV PCR^a^	PACE^b^	AGID^c^	Pestivirus PCR^a^	BDV PCR^a^
A	Small and hairy	33.1	24.5	31.1	24.1	1.04	2	38.1	27.5
B	Small and hairy	32.4	22.9	31.7	23.8	1.13	3	39.3	26.8
C	Small and hairy	29.8	23.2	28.5	20.7	1.06	3	Negative	28.5
D	Small and hairy	Negative	Negative	Negative	Negative	Negative	2	Negative	Negative
E	Hairy	30.9	23.6	29.6	21.3	0.85	2	32.2	24.8
F	Hairy	Negative	30.0	Negative	34.0	0.98	3	Negative	28.1
G	Hairy	Negative	Negative	Negative	Negative	Negative	2	Negative	Negative
H	Hairy	Negative	Negative	Negative	Negative	Negative	2	Negative	34.3
I	Hairy	36.3	28.5	Negative	34.4	0.95	>3	Negative	31.5
J	Hairy	30.3	23.6	29.3	21.8	1.45	2	34.2	28.6
K	Hairy	28.7	22.3	27.4	20.0	1.02	2	Negative	29.4
L	Hairy	31.9	23.5	29.7	22.5	1.24	1	Negative	26.6

^a^ Real‐time reverse transcription polymerase chain reaction (qRT‐PCR): Cycle threshold (Ct) values <40 are considered to be positive.

^b^ Pestivirus antigen capture ELISA (PACE): Corrected Optical Density (OD) values ≥0.4 are positive.

^c^ Agar gel immunodiffusion assay (AGID): Positive results are graded 1 to >3 depending on the strength of reactivity relative to the positive control.

Samples were tested to detect viral RNA in both pan‐reactive and Border disease virus‐specific real‐time reverse transcription polymerase chain reaction (qRT‐PCR) assays. Samples were also tested to detect pestivirus antigens in serum using a pestivirus antigen capture ELISA (PACE). Antibody levels in serum were determined using a pan‐pestivirus agar gel immunodiffusion assay (AGID). Ewe cohorts include 2014 (Lambs A and B), 2015 (Lambs C to G) and 2017 (Lambs H‐L) born ewes.

#### Detection of and monitoring of PI lambs from maiden Composite ewes

Based on the initial results from examining the progeny of the mature Composite ewes, lambs with abnormal coats were preferentially selected during marking and weaning. Animals were classified as normal appearance (N), extra hair on face and legs (H1) or very hairy—generalised (H2). A total of 273 clotted blood samples were collected from 2‐ to 8‐week‐old lambs (n = 159; Paddock A and B) and 12‐week‐old lambs (n = 114; Paddocks C‐F) between early October and early November 2019 (Table 5).

**Table 5:** Comparison of pestivirus antigen capture (PACE) results for persistently infected lambs from maiden Composite ewes at Property 1, with different coat characteristics

Paddock	No. sampled	PACE^a^ positive				PACE^a^ negative				
		H2	H1	N	Total	H2	H1	N	Total
A	129	47	19	3	69	0	21	39	60
B	30	11	0	1	12	0	2	16	18
C	33	13	13	0	26	0	6	1	7
D	29	10	18	0	28	1	0	0	1
E	28	5	23	0	28	0	0	0	0
F	24	8	16	0	24	0	0	0	0
Total	273	94	89	4	187	1	29	56	86

^a^ Pestivirus antigen capture ELISA (PACE): Corrected Optical Density (OD) values ≥0.4 are positive.

Lambs with abnormal coats were preferentially selected for sampling. Animals were classified as H2 (very hairy – generalised), H1 (extra hair on face and legs) or N (normal appearance). Lambs from Paddock A and B were aged 2–8 weeks of age and lambs from Paddocks C–F were 12 weeks old.

#### Monitoring PI lambs and naïve in contact ewes

A selection of PI lambs (n = 120) aged approximately 6–10 weeks with coats classified as H1 or H2 was retained at weaning. They were placed with a group of eight‐month‐old presumptively normal lambs (n = 3400) in a confinement feeding area for 4 weeks. Clotted blood samples were collected from the normal eight‐month‐old ewes (n = 30) to monitor the extent of pestivirus transmission before joining. A subset (n = 14) of surviving PI lambs was then moved to a neighbouring property and sampled at approximately 3‐monthly intervals until 13–15 months of age (Table 6).

**Table 6:** Sequential virology results for surviving persistently infected lambs from maiden Composite ewes at Property 1

Age	2–3 months			5–6 months				9–11 months			13–15 months			
Lamb	PACE^a^	BDV PCR^b^	AGID^c^	PACE^a^	BDV PCR^b^	AGID^c^	BDV VNT^d^	PACE^a^	BDV PCR^b^	AGID^c^	PACE^a^	BDV PCR^b^	AGID^c^	BDV VNT^d^
1	0.58	30.0	1	0.87	27.0	Negative	8	1.9	27.8	Negative	1.96	30.2	Negative	8
2	1.12	22.2	Negative	0.88	25.9	Negative	<4	0.7	25.4	Negative	0.83	26.7	Negative	<4
3	0.60	23.5	1	0.83	23.5	Negative	<4	0.9	24.9	Negative	0.77	28.3	Negative	<16^f^
4	0.83	24.5	1	0.86	25.5	Negative	<4	1.3	26.5	Negative	1.13	28.4	Negative	<4
5	0.43	23.4	3	0.96	26.9	Negative	<4	1.2	27.1	NT^e^	0.97	29.7	Negative	<4
6	0.82	22.4	1	0.97	27.3	Negative	<4	1.3	26.4	Negative	0.74	25.4	Negative	<4
7	0.85	26.3	2	0.86	24.2	Negative	<4	1.1	27.7	Negative	0.80	29.7	1	<4
8	0.94	26.3	1	0.90	24.6	Negative	<4	0.9	27.7	Negative	0.91	27.8	Negative	<4
9	0.95	23.8	Negative	1.07	25.7	Negative	<4	1.2	24.5	Negative	1.10	24.8	Negative	<4
10	0.84	24.0	2	0.78	23.9	Negative	<4	1.0	24.7	Negative	0.85	25.8	Negative	<4
11	1.07	22.6	1	0.59	25.2	Negative	<4	0.9	27.4	Negative	0.96	29.2	>3	<4
12	0.87	23.9	Negative	0.91	27.2	Negative	<4	1.2	27.7	Negative	0.89	27.7	Negative	<4
13	0.88	22.5	1	0.91	25.3	Negative	<4	1.0	27.0	Negative	1.02	26.6	Negative	<4
14	1.18	24.6	1	0.93	26.0	Negative	<4	1.2	26.1	Negative	0.91	27.9	Negative	4

^a^ Pestivirus antigen capture ELISA (PACE): Corrected Optical Density (OD) values ≥0.4 are positive.

^b^ Real‐time reverse transcription polymerase chain reaction (qRT‐PCR): Cycle threshold (Ct) values <40 are considered to be positive.

^c^ Agar gel immunodiffusion assay (AGID): Positive results are graded 1 to >3 depending on the strength of reactivity relative to the positive control.

^d^ Virus neutralisation test (VNT): Titres <4 are considered to be negative.

^e^ Not tested (NT).

^f^ Not suitable for testing at lower dilutions.

Lambs were sampled at 3‐monthly intervals until 13–15 months of age. Samples were tested to detect pestivirus antigens in serum using a pestivirus antigen capture ELISA (PACE). Samples were also tested to detect viral RNA in the Border disease virus‐specific real‐time reverse transcription polymerase chain reaction (BDV PCR) assays. Antibody levels in serum were determined using a pan‐pestivirus agar gel immunodiffusion assay (AGID) and a Border disease virus neutralisation test (VNT).

### History—Property 2

This property ran a mixed commercial Composite operation with 6500 ewes and 40 beef cattle across two farms (Farm A and Farm B), approximately 10 kilometres apart. Before joining in 2019, maiden ewes had moved from Farm B with cattle in adjacent paddocks, to Farm A. No cattle were held at Farm A and no reproductive concerns were documented in the cattle. Historically, a small number of small and hairy lambs had been observed at Farm B. Animal introductions to Farm A included rams from two external sources and replacement ewes from two different external sources in the preceding 2 years. All maiden ewes were vaccinated against a wide range of common pathogens (Ovilis Campyvax (Coopers©), Glanvac® 6 B12, Gudair and Scabiguard (Zoetis©)).

In March 2019, maiden ewes were joined at 7–8 months of age for 8 weeks with rams included at a rate of 1.6% for 6 weeks and 3.7% for the final 2 weeks. Ewes were then run on either improved or native pastures, depending on their pregnancy status, and those with lower body condition score (BCS) were supplementary fed in the paddock to maintain a BCS of 3. In June 2019, ultrasound pregnancy scanning on Farm A found foetuses apparently undergoing resorption in maiden ewes (n = 9), initiating an investigation. No follow‐up scanning was reported. However, this followed a two‐year history of suboptimal scanning results where previous diagnostic investigations in 2018 on Farm B excluded direct involvement of toxoplasmosis and pestiviruses by serology but identified mildly elevated *Campylobacter fetus* subspecies *fetus and jejuni* antibody titres, considered to be due to vaccination.

### Specimen collection—Property 2

#### Flock seroprevalence

In late June 2019, following the ultrasound scanning results from Farm A that indicated possible foetal resorption and abortion, clotted blood samples were collected from affected maiden Composite ewes (n = 9). Clotted blood samples were also collected from heifers (n = 10) in late July 2019 at Farm B, where the maiden ewes were held before being moved to Farm A (Table 7). Subsequently, to assess the pestivirus status of Farms A and B, there was extensive collection of sera, taking into account the age (born 2011–2017 and 2018), breed (Composite) and sex structure (ewes and rams) of animals present (Table 8).

**Table 7:** Initial pestivirus serology results from maiden Composite ewes and cattle located at Property 2

Sample description	AGID^a^	BVDV VNT^b^	BDV VNT^b^
Affected ewe – 1	Negative	<8^c^	<4
Affected ewe – 2	3	64	4096
Affected ewe – 3	2	64	1024
Affected ewe – 4	3	32	4096
Affected ewe – 5	2	32	1024
Affected ewe – 6	Negative	<4	<4
Affected ewe – 7	3	64	4096
Affected ewe – 8	Negative	<8^c^	<4
Affected ewe – 9	3	32	2048
Cattle – 1	Negative		
Cattle – 2	3	512	4
Cattle – 3	Negative		
Cattle – 4	Negative		
Cattle – 5	Negative		
Cattle – 6	3	512	32
Cattle – 7	Negative		
Cattle – 8	Negative		
Cattle – 9	3	128	8
Cattle – 10	Negative		

^a^ Agar gel immunodiffusion assay (AGID): Positive results are graded from 1 to >3 depending on the strength of reactivity relative to the positive control.

^b^ Virus neutralisation test (VNT): Titres <4 are considered to be negative NT.

^c^ Sample not suitable for testing at lower dilutions.

Affected Composite ewes were located at Farm A and had abnormal scanning results including resorbing foetuses. Cattle were located at Farm B and were clinically normal. All samples were tested in a pestivirus pan‐reactive agar gel immunodiffusion assay (AGID). Samples giving positive results in the AGID were tested in virus neutralisation (VN) tests for Border disease virus (BDV) and bovine viral diarrhoea virus (BVDV).

**Table 8:** Flock seroprevalence of pestivirus antibodies at Farms A and B on Property 2 as determined by agar gel immunodiffusion assay (AGID)

Cohort	Breed	Population	Prevalence % (95% CI)	AGID^a^					
				Negative	1	2	3	>3	Total
Farm A									
Mixed	Composite ewes	1855	51 (40–62)	37	10	8	16	4	75
2018	Composite ewes	1007	58 (42–71)	17		4	19		40
Total		**2862**	**53 (44–62)**	**54**					**115**
Mixed	Composite rams	42	70 (40–89)	3		4	3		**10**
Total		**42**	**70 (40–89)**	**3**					**10**
Farm B									
Mixed	Composite ewes	2540	2 (0–6)	118	2				120
2018	Composite ewes	810	0 (0–9)	41					41
Total		**3350**	**1 (0–4)**	**159**					**161**
Mixed	Composite rams		0 (0–28)	**10**					10
2018	Composite rams		20 (6–51)	**8**		2			10
Total			**10 (3–30)**	**18**		**2**			**20**

^a^ Agar gel immunodiffusion assay (AGID): Positive results are graded 1 to >3 depending on the strength of reactivity relative to the positive control.

Samples were collected from both older (2011–2017) and maiden (2018) Composite ewes and ram groups from both Farms A and B.

#### Detection of PI lambs

To detect PI lambs at Farm A, clotted and EDTA‐treated blood samples and tonsillar swabs were collected from suspect lambs at both marking and weaning (n = 31) at approximately 6–8 weeks of age and 12–14 weeks of age respectively.

### Necropsy and histopathology

A full range of fresh and fixed tissues was collected at necropsy of foetuses that arrived at the laboratory in a suitable condition. Histological samples were fixed in 10% buffered formalin for a minimum of 24 hours, embedded in paraffin wax, then sectioned and stained by standard methods.

### Microbiology

A comprehensive range of tests for microorganisms that could potentially affect reproductive performance was conducted and included aerobic culture; selective cultures for *Salmonella sp*., *Listeria sp*. and *Campylobacter sp*; serology for Campylobacter spp. (serum agglutination tests), Chlamydia (complement fixation test); *Coxiella burnetii* enzyme‐linked immunosorbent assay (ELISA) and *Toxoplasma gondii* (latex agglutination test). Polymerase chain reaction (PCR) assays were also conducted for *Chlamydia sp*. and *Coxiella burnetii*.

### Virology

#### Pestivirus antigen detection

Serum, plasma, thoracic and pericardial fluid and supernatant from 10% homogenates of lung and spleen in phosphate‐buffered saline were tested using a commercial pestivirus antigen capture ELISA (PACE) (IDEXX BVDV Ag/Serum Plus, IDEXX Laboratories, Switzerland) which was run according to the manufacturer's instructions.

#### Pestivirus real‐time reverse transcription polymerase chain reaction PCR (qRT‐PCR) assays

Prior to nucleic acid extraction from tissues including brain, lung and spleen, a freshly cut surface was swabbed with a dry, cotton‐tipped swab which was placed and left in 3 mL of viral transport medium (phosphate‐buffered gelatine saline, PBGS). Total nucleic acids were then extracted from 50 μL of either serum, pericardial or thoracic fluid or PBGS or 25 μL of EDTA‐treated whole blood using the MagMax‐96 viral RNA isolation kit (Applied Biosystems) and a magnetic particle handling system (Kingfisher‐96, Thermofisher) according to the manufacturer's directions. qRT‐PCR reactions were prepared using 5 μL of nucleic acid in 25 μL reactions using the AgPath‐ID one‐step RT‐PCR kit (AM1005, Applied Biosystems) and run according to the cycling conditions specified for the mastermix using a 7500 Fast Real‐Time PCR System (Applied Biosystems) run in standard mode for a total of 45 cycles. Pestivirus RNA was detected using a pan‐pestivirus reactive qRT‐PCR^28^ or a BDV‐specific qRT‐PCR^29^ and the results compared with assess sensitivity. An exogenous internal control was included in the sample lysis buffer for subsequent extraction, amplification and detection in each reaction as described by Gu et al.^30^ The fluorescence threshold was set manually at 0.05 and background was automatically adjusted (ABI 7500 software v.3). qRT‐PCR results were expressed as cycle threshold (Ct) values and classified as negative if no amplification was observed after 45 cycles.

#### Pestivirus serological tests

Samples were initially screened with a broadly reactive pestivirus agar gel immunodiffusion (AGID) assay.^31^ Results were expressed as negative when no precipitin line was detected and scores of 1 to >3 assigned to positive samples with increasing levels of reactivity. To identify infection with a specific pestivirus species, virus neutralisation tests (VNTs) were used^31^ with the inclusion of either BDV or BVDV as the test virus. The BDV VNT was based on the Australian reference strain “Clover Lane” (X818), whereas the BVDV VNT utilised the Australian “Trangie” strain.

### Animal ethics approval

As sampling was undertaken for diagnostic and disease control purposes, animal ethics approval was not required.

## Results

### Property 1

#### Initial reproductive impact: maiden Composite ewes

In 2019, ultrasound pregnancy scanning results in maiden Composite ewes showed that of the 3500 joined, only 2448 (69.9%) were found pregnant at the initial scan at approximately 85–90 days of gestation (range 67–108 days). Only 1922 lambs were marked at approximately 2–6 weeks of age, representing a loss of more than 500 lambs. Only 54.9% of ewes joined produced lambs that were present at marking, a reduction of a further 15% from scanning to marking. In contrast to 2019, of the 4037 maiden Composite ewes joined in 2018, 3378 (83.7%) were shown to be pregnant at the initial scan, and 2814 lambs (69.7% of ewes joined) were marked.

#### Foetal infections

The first two foetuses submitted were undergoing resorption, demonstrated by thin foetal membranes, watery brown allantoic and amniotic fluids and brown to purple foetuses with friable internal organs. A sparse pure growth of *Escherichia coli* (nonhaemolytic) was isolated from one placenta and was not considered to be clinically significant. No other microorganisms or viruses were identified. Complete histological examination was affected by marked autolysis. A moderate, multifocal, necrotising and neutrophilic, subacute placentitis was observed in one of the foetuses.

The next aborted foetus was in an advanced state of autolysis. Swabs of the abdominal tissues and placenta gave weak reactivity in the pan‐pestivirus qRT‐PCR (Ct 35.63 and 34.63 respectively) and in the BDV‐specific qRT‐PCR (Ct 32.37 and 31.71 respectively). Pestivirus antibody was also detected in a sample of foetal fluid (AGID score 2) from this foetus.

The 10 full‐term dead lambs collected from maiden (2018) ewes were received in two laboratory submissions (Table 1; Lambs 1–4 and 5–10).

The first 4 lambs were classified as perinatal deaths based on a lack of evidence of walking or suckling; however, the lung tissue from all four foetuses floated in formalin, suggesting they were born alive but died soon afterwards.^32^ The subsequent submission consisted of three fresh (Lambs 5–7) and three frozen (Lambs 8–10) lambs. Of these, four were classified as perinatal deaths and two as stillbirths (lung tissue sank in formalin). A single foetus (lamb 5) (perinatal death) had evidence of walking and suckling (Table 1).

BDV was detected in seven of the 10 lambs by several diagnostic assays. There was evidence of BDV infection in a wide range of tissues and body fluids as detected by the BDV‐specific qRT‐PCR. Although Lamb 5 had ingested colostrum, its BDV status was clearly established by the BDV qRT‐PCR. There was reduced reactivity in the PACE, probably due to competition with the colostral antibodies. The highest virus concentrations (lower Ct values) were usually found in pericardial or thoracic fluid. In contrast, approximately half (16 of 35) of the samples in which BDV RNA was detected gave negative results in the pan‐reactive qRT‐PCR. The PACE detected BDV antigen in the spleen of all seven animals that were positive in the BDV qRT‐PCR and in the pericardial fluid of six of these lambs. Only three of the seven BDV infected lambs had straight wool that would be regarded as “hairy”. Histologically, the skin demonstrated variably sized hair follicles; however, no diagnostic conclusions could be made. There was no evidence of bacterial infection although samples from the first four lambs were unsuitable for culture due to transport delays.

#### Flock seroprevalence

Of the 30 blood samples submitted from maiden Composite ewes 28/30 (93%) were positive in the pestivirus AGID (Table 2). All of the AGID positive samples had very high titres in the BDV VNT. When tested in the BVDV VNT, the sera from the maiden ewes considered to have resorbing foetuses showed much lower antibody titres, consistent with infection with BDV rather than BVDV (Table 2). There was no apparent difference in AGID results or BDV VN titres between groups that were identified to have resorbing foetuses, to be nonpregnant or were unaffected pregnant ewes. The two maiden crossbred ewes that gave negative results in the AGID also gave negative results in both the PACE and the BDV VN assays.

The 30 blood samples from the maiden Composite ewes also gave negative results in assays for Leptospira (*L. hardjo* and *L. pomona*) and *T. gondii* and only two of the samples were seropositive in a *C. burnetii* antibody ELISA. All 30 sera reacted in the *Campylobacter fetus fetus* serum agglutination test and 14 were positive in the *Campylobacter jejuni* assay. These results were considered to be associated with the history of recent vaccination before joining. The cross‐sectional serological assessment of the remaining animals at Property 1 revealed strongly contrasting differences between the cohorts of Composite ewes and the Merino ewes. The prevalence of antibodies detected by AGID in the Composite ewes ranged from 75% to 100% (Table 3). Although strong reactors (score 3 or >3) in the AGID were detected in the 2015, 2016 and 2017 Composite cohorts, the highest proportion of strongly reacting animals were found in the 2018 maiden Composite cohort with four of the 30 (13%) animals giving an AGID score of >3. There was a moderate‐to‐high seroprevalence in the Dorset (71–93%) and Composite (21–63%) breeding rams, and no seropositive animals in the recently purchased (2019) Dorset and Composite weaner rams. Unlike the Composite ewes, there was limited evidence (5%–15%) of seropositivity in older (2014–15) Merino ewes and Merino rams (10%), and no seropositive animals in the more recent (2016–17) Merino ewe groups (Table 3).

#### Examination of progeny of mature ewes

An abnormal “hairy” coat was the predominant observation in affected lambs on Property 1, with “shaking” lambs only observed occasionally. Lambs that were the progeny of mature Composite ewes were selected for BDV testing based on body size and ‘hairy’ coat appearance. Only two suspect (“slightly hairy”) Merino lambs were found at marking despite inspecting lambs from more than 5000 Merino ewes. No evidence of BDV infection was found in these two lambs. Ten of the 12 suspect lambs from mature Composite ewes were shown to be infected with BDV (Table 4). While BDV was not detected in the samples from the remaining two lambs, both were seropositive, indicating that either they were infected *in utero* or were the progeny of ewes that had been infected. Of the three sample types that were compared with establish the preferred sample for ongoing large‐scale investigations, based on lower Ct values, superior BDV qRT‐PCR results were most often found in serum compared with EDTA‐treated whole blood or tonsillar swabs. While there was overall agreement for the detection of BDV RNA using all three sample types, only very low levels of BDV RNA were detected in the tonsillar swab of one lamb without a detection in blood.

#### Correlation between clinical presentation and PI status of lambs from maiden Composite ewes

The correlation between the extent of changes to the coat (‘degree of hairiness’), body size and persistent infection with BDV was reinforced when the progeny of the 2018 maiden ewes were examined and serum samples were tested with the PACE (Table 5). All but one lamb (94/95) that was described as “very hairy, generalised” (H2) was positive in the PACE. Similarly, 89 of the 118 lambs that had ‘extra hair on face and legs’ (H1) were also positive in the PACE. In contrast, only a small number (4/60) of lambs with a normal appearance were positive in the PACE.

#### Monitoring PI lambs and naïve exposed ewes

One hundred and twenty animals that were classified as either H1 or H2 and were also PACE positive were retained at weaning. Of these, only 18% (21 lambs) survived until sampling at approximately 5–6 months of age. However, the impact of dry seasonal conditions on the survival of these animals is uncertain. The 21 lambs were in a body condition score of 1.5–2.0 (thin) when moved to another property for ongoing monitoring. They were held on an established lucerne paddock, and their fleece shorn and were sampled at approximately 5–6 months (n = 21), 9–11 months (n = 17) and 13–15 months (n = 16). Of these 21 lambs, 14 were sampled at all four time points (Table 6). At 9 months of age, all animals were growing normally, weighing approximately 40‐45 kg, except for a single animal (Table 6 – Lamb 8) that was half the size of the remaining cohort. Once shorn, the lambs showed no further signs of the characteristic “hairy” appearance in their fleece.

The prevalence of antibodies detected by AGID was moderate in naïve eight‐month‐old Composite ewes (17/30; 56.7%) that were managed intensively for 4 weeks with the 120 PI lambs in a feedlot‐like situation before joining. The remaining 13 lambs that did not seroconvert in the time that they were held with the PI lambs were tested in the PACE with negative results.

### Property 2

#### Initial reproductive impact: maiden Composite ewes

In 2019, ultrasound pregnancy scanning results in maiden Composite ewes showed that, of 1185 ewes scanned at Farm A, 999 (84.3%) were found to be pregnant at the initial scan at approximately 85–90 days of gestation (range 47–104). Only 9 ewes were identified as having foetuses suspected of undergoing resorption. Of the expected 1434 lambs (121% of maiden ewes scanned in lamb) 1203 were marked at approximately 2–11 weeks of age. On Farm B, only 923 of 1210 (76.3%) maiden ewes were found to be pregnant at the initial scan. Of the expected1379 lambs (114% of ewes scanned) only 921 lambs (76.1% of ewes scanned) were marked at approximately 2–11 weeks of age.

These results follow on from 2018, where ultrasound pregnancy scanning of a subset of a maiden Composite ewe flock on Farm B found that, of 257 ewes scanned, only 207 (80.5%) were pregnant, including 25 ewes with an abnormal ultrasound image. A diagnostic investigation in 2018 involving 12 ewes found no serological evidence of pestivirus or toxoplasma infection and poor reproductive performance was attributed to inadequate nutrition. In contrast to Property 1, to build flock numbers on Property 2 all maiden Composite ewes were joined, including some with an estimated joining weight of 35 kilograms compared with the average weight of mature adult ewes of 85–90 kg. In addition, in the month before joining (March 2019) on Farm A, the maiden Composite ewes had a significant *Haemonchus contortus* burden with some deaths, suggesting multiple factors may have impacted the reproductive outcomes of the maiden ewes on Property 2 in 2019.

#### Flock seroprevalence

Of the nine blood samples submitted from maiden ewes at Farm A to investigate the reproductive losses detected at scanning, 6/10 (67%) were seropositive in the pestivirus AGID and 3/10 (30%) of the blood samples submitted from the cattle at Farm B where the maiden crossbred ewes had originally been held were seropositive. Further testing in the VNT revealed results consistent with the cattle being previously exposed to BVDV and the maiden ewes being previously exposed to BDV (Table 7).

The cross‐sectional serological assessment of sheep on the two farms indicated a marked difference in BDV seroprevalence. At Farm A, there was a moderate seroprevalence of pestivirus antibody (51–70%) in Composite maiden ewes, mixed‐age ewes and rams (Table 8). In contrast there was little evidence of BDV infection (<5%) in mixed‐age and maiden Composite ewes and only two of the 2018 Composite rams held at Farm B.

#### Investigation of PI lambs

The number of suspect PI lambs on Property 2 was significantly less than on Property 1 and as a consequence, no grading system was implemented. Suspect lambs were only sampled if they were small and/or hairy lambs with goat‐like hair.

**Figure 1:**
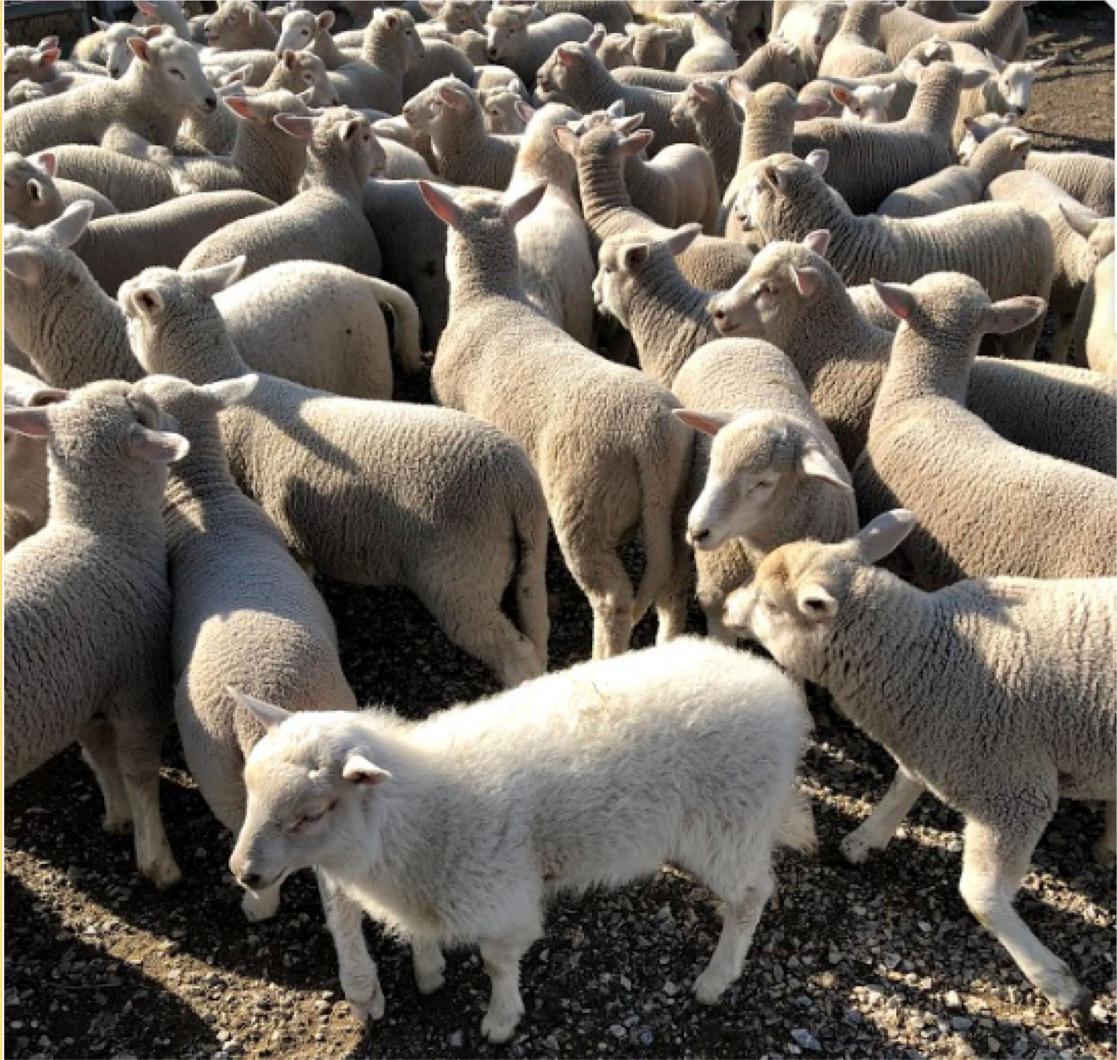
A 3‐month‐old, persistently infected lamb (H2) at lamb marking on Property 2. Animals classified as H2 had a very hairy coat over the whole body.

At Farm A, many lambs were born with hairy coats and were easily distinguished from other lambs (Figure 1). Thirty‐one suspect lambs were available for paired testing at marking and weaning. Twenty lambs were shown to be PI animals, a single lamb seroconverted and gave negative results in the PACE and qRT‐PCR at weaning and 10 were negative in all assays at both time points. Serum from all PI lambs gave positive results in the PACE at both time points, and both serum and tonsillar swabs were positive in the PCR assay at the same time points (Table 9). The reactivity detected in the AGID at marking, consistent with maternal antibodies, was no longer present at weaning in all but two lambs (Table 9). At Farm B, very few lambs with hairy coats were observed, and all suspect lambs sampled at marking (n = 3) gave negative results in all assays and were not sampled again.

**Table 9:** Border disease virology results for samples collected from persistently infected Composite lambs at marking and weaning at Farm A, Property 2

Lamb	Marking				Weaning			
	Serum			Tonsillar swab	Serum			Tonsillar swab
	PACE^a^	BDV PCR^b^	AGID^c^	BDV PCR^b^	PACE^a^	BDV PCR^b^	AGID^c^	BDV PCR^b^
1	0.62	23.2	3	26.8	0.93	26.6	1	29.5
2	0.76	28.7	2	31.9	1.18	27.3	Negative	31.0
3	0.75	22.6	2	32.9	1.36	27.4	Negative	30.9
4	0.72	27.4	3	32.3	0.93	27.7	Negative	31.8
5	0.62	23.8	1	31.7	0.90	28.0	Negative	31.9
6	0.89	23.7	1	33.2	1.18	28.1	Negative	26.7
7	0.80	24.5	1	32.5	1.10	28.2	Negative	32.3
8	0.72	21.6	2	27.7	1.20	28.3	Negative	29.6
9	0.72	23.8	2	31.9	1.13	28.3	Negative	31.6
10	0.74	24.8	1	32.7	1.02	28.5	Negative	31.2
11	0.91	23.3	1	32.0	1.36	28.9	Negative	28.8
12	0.56	23.8	3	34.6	0.91	28.9	1	31.8
13	0.67	24.4	3	31.9	1.14	29.1	Negative	32.2
14	0.53	24.8	3	32.2	1.13	29.6	Negative	33.4
15	0.75	26.9	1	35.1	1.31	29.6	Negative	33.1
16	0.79	27.0	1	34.1	1.12	29.9	Negative	31.6
17	0.68	22.7	2	33.0	1.24	30.3	Negative	31.5
18	0.74	26.0	2	31.0	1.02	30.4	Negative	31.7
19	0.54	27.5	2	29.8	0.94	30.9	Negative	31.9
20	3.49	27.4	2	36.9	3.81	32.2	Negative	32.0

^a^ Pestivirus antigen capture ELISA (PACE): Corrected Optical Density (OD) values ≥0.4 are positive.

^b^ Real‐time reverse transcription polymerase chain reaction (qRT‐PCR): Cycle threshold (Ct) values <40 are considered to be positive.

^c^ AGID: Score Negative to >3; agar gel immunodiffusion assay (AGID): Positive results are graded 1 to >3 depending on the strength of reactivity relative to the positive control.

Composite lambs were inspected at marking when approximately 2 months of age and again at weaning when 3 months of age. Lambs with abnormal coats were preferentially selected for sampling and tested to detect pestivirus antigens in serum using a pestivirus antigen capture ELISA (PACE) and viral RNA in the Border disease virus‐specific real‐time reverse transcription polymerase chain reaction (BDV PCR) assay. Antibody levels in serum were determined using a pan‐pestivirus agar gel immunodiffusion assay (AGID).

## Discussion

In contrast to the well documented economic impacts of BVDV on the Australian beef cattle industry, estimated in 2022 at $67.9 M annually, estimates of the financial losses associated with BDV infection in Australian sheep are limited.^33^ BDV infection and disease have been considered to be uncommon to rare in Australian sheep flocks, although there is limited supportive virological evidence.^34^, ^35^, ^36^, ^37^, ^38^, ^39^ Early serological studies often used BVDV, rather than BDV when detecting neutralising antibodies in sheep samples, potentially resulting in antibody titres and seroprevalence being underestimated.^37^, ^40^ In the early 1990s, an extensive serological survey of Australian Merino studs did not detect any evidence of BDV infection (Shannon, AD and Kirkland, PD, unpublished data) and more recently a study that examined the seroprevalence of BDV in sheep in South Australia, reported an individual animal prevalence of 0.35% (3/875 sheep from 29 farms) with the positive animals all from the same farm.^38^

In contrast to past observations, this study highlights the pathogenic potential of BDV. The profound production losses experienced highlight the need for robust seroprevalence surveys, to better understand both the true prevalence, and the potential economic impact BDV has on the Australian sheep industry. Using the long‐term lambing rates for the properties under investigation, it is estimated that more than 500 lambs were lost on both Property 1 and Property 2, without considering possible losses before the first scanning for pregnancy.

The low seroprevalences reported in previous studies are a marked contrast to the serological profiles found on both properties in the current investigation. Poor reproductive performance, including conception failure and the detection of foetal resorption during ultrasound scanning in early pregnancy provided the key catalyst for further investigation. In the absence of foetal material, which is needed for definitive confirmation of disease due to BDV, demonstrating that these early losses are due to BDV infection can be difficult. However, maternal serology is extremely useful. A negative result will exclude pestivirus involvement, whereas positive results indicate that pestivirus infection should be considered further. The high pestivirus seroprevalence detected using the AGID in maiden ewes from both Property 1 and Property 2 (Farm A) prompted further testing by BDV‐specific VNT to establish involvement of BDV rather than BVDV. Further investigation into the flock seroprevalence on Property 1 highlighted a higher seroprevalence in younger animals linked with the occurrence of disease and in Composite ewes compared with Merino ewes. In this instance the Merino sheep were managed separately from the Composite sheep and in paddocks separated from the rest of the property by a road. The lower prevalence in the oldest age groups is consistent with the infections occurring in recent years and provides evidence that segregation of animal groups based on age or by management can impact heavily on the transmission of pestiviruses. As antibodies to pestiviruses generally persist for many years, had there been extensive mixing of age cohorts, a much higher prevalence in the older sheep would be expected. On Property 2, Farm A had a much higher prevalence in Composite ewes than Farm B. Furthermore, the recent management practices of joining maiden Composite ewes at 7–9 months of age have probably had a profound impact on BDV transmission. At this early joining age there is limited opportunity to develop immunity after the loss of maternal antibodies and before joining. There is also a greater likelihood of there being a larger number of surviving PI lambs. When these factors are combined with the intensive management and confinement feeding practices implemented during drought conditions to provide optimal nutrition and growth of maiden ewes before joining, there is greater potential for large‐scale BDV transmission.

The presence of a hairy birth coat and shaking lambs has been synonymous with BDV infection and these PI lambs represent the most potent ongoing source of infection.^3^ Nevertheless, little is known about the rate of BDV transmission when susceptible sheep are held in confinement with PI lambs. In a recent study, it was shown that when 66 naïve sheep were held in confined conditions with two PI lambs, less than 5% (3 of 66) had seroconverted after 15 days.^41^ This is in marked contrast to BVDV transmission where infection rates of more than 50% have been reported in 24 h when there is close contact between PI calves and susceptible cattle.^42^ In the current study, we observed seroconversion of 56.7% of a group of 8‐month‐old Composite ewes exposed to a group of PI lambs (n = 120, a ratio of 3.6%) in a feedlot‐like situation for 1 month before joining. Despite the extremely high number of PI lambs included and the relatively high seroconversion rate observed, almost half of the ewes were still susceptible after 4 weeks. This outcome raises questions related to the role in virus transmission of surviving PI lambs and their virus excretion levels compared with the extremely high virus loads in an aborted foetus and the associated fluids and membranes or lambs that are either stillborn or die soon after birth.

The BDV incidents described in these diagnostic investigations each show characteristic features of pestivirus infections and are supported by extensive laboratory confirmation. The large number of PI lambs that were born provided a unique opportunity to compare the clinical presentation with laboratory results. There was an extremely high level of agreement between the lambs' physical appearance (‘hairiness’) and their infection with BDV. The short life expectancy of PI lambs is considered typical and contributes to the variable transmission of BDV in a population as the number of PI lambs declines.^12^ However, from a group of 120 PIs identified at weaning on Property 1, 14 were still alive 12 months later. Abnormal coat was the predominant observation on both properties suggesting that the PI lambs that show abnormal hair coat presumably have limited virus damage to the central nervous system. Experimental transmission studies have shown that the occurrence of neurological disease is a characteristic of the virus strain rather than the stage of gestation at which infection has occurred.^12^, ^13^, ^14^ The lack of cases of neurological disease and the overwhelming presence of hairy coats is a consistent feature of the virus strains involved in these outbreaks.

The results of this study also illustrate the benefits of both basic procedures and current technology for pestivirus diagnosis. While the AGID is considered to be a basic test, it is robust and tolerates poor quality samples from a range of sources very well. Samples such as pericardial fluid and heart blood from an aborted or stillborn lamb are frequently unsuitable for testing in a VNT but an AGID can yield valuable results.

The index case for Property 1, for which BDV infection was confirmed and led to more intensive investigation, was an aborted foetus in which a low level of BDV RNA was detected by PCR. In the past, virus isolation was the only method that could be used to confirm BDV infection in a foetus.^3^ In this instance, virus isolation could not be carried out due to the poor condition of the specimens and, if there was viable virus present, it is likely that, based on the low level of RNA detected, there would not have been sufficient infectious virus to be detected by culture even if the effects of contamination were avoided. The BDV‐specific qRT‐PCR was the most useful assay, detecting viral RNA in a wide range of tissues and body fluids. The highest concentrations (lower Ct values) were usually found in pericardial fluid. In contrast a proportion of the samples in which BDV RNA was detected gave negative results in the pan‐reactive pestivirus qRT‐PCR.

From a rapid diagnostic perspective, the PACE assay was shown to have adequate sensitivity and breadth of reactivity to allow the detection of BDV antigens in most of the PI lambs and foetal material. In this study, follow‐up testing to confirm PI status was only undertaken on a limited number of animals. However, these lambs were preferentially selected on the basis of size and the nature of their coat. Hairy birth coat is the product of infection in early to midgestation rather than in late pregnancy. Therefore, it is highly likely that these were PI lambs. Additionally, acutely infected animals, whether infected in late gestation or postnatally, are unlikely to have antigen levels sufficient to give significant reactivity in the PACE. While the PACE performed extremely well, results should be monitored closely and supplemented with alternative assays to ensure that colostral antibody does not suppress reactivity and give false negative results.

Appropriate sample selection is always important to optimise laboratory confirmation of an infectious disease. In addition to blood and tissues such as brain, lung and spleen, pericardial fluid was a useful sample from aborted and stillborn lambs. As well as detection of viral antigens or RNA, pericardial fluid also allows serology to be undertaken to identify animals that have been infected late in gestation or with other agents.

Paired samples from suspect PI lambs at marking and weaning on Property 2 highlight the importance of considering the impact of maternal antibodies when testing serum from young lambs in the PACE assay. At marking (approximately 2 months of age), all lambs gave positive results in both the PACE and AGID. At weaning (approximately 3 months of age), all lambs continued to give positive results in the PACE, with stronger reactivity, but only two of the 20 lambs remained weakly seropositive. In suspect PI lambs, serum also provides the opportunity for detection of viral antigens, either by PCR or PACE, and allows serology to be undertaken on the same sample.

## Conclusions

These investigations have demonstrated that although BDV infection has been considered to be uncommon in the Australian sheep population based on limited historic evidence, there are circumstances in which BDV transmission can increase to a level where significant reproductive loss can occur. Given there have been industry trends towards joining of maiden ewes at a younger age, often in conjunction with intensive feeding to accelerate growth, or to maintain body weight in drought, there is a likelihood that BDV infections will be encountered more frequently. BDV infection should be considered when early pregnancy failures are detected by ultrasound scanning or where ewes with confirmed pregnancies fail to produce a live lamb. The investigations described in these outbreaks highlight the range of samples to be collected and the selection of appropriate assays to achieve a reliable diagnosis.

Considerable care should be exercised if confined management practices and joining are undertaken in extended drought conditions. To better understand the economic impact that BDV may be having on the Australian sheep population, intensive serological surveys in the major sheep‐producing states are recommended.

## Conflicts of interest and sources of funding

The authors declare no conflicts of interest or sources of funding for the work presented here.

## Data Availability

The data that support the findings of this study are available from the corresponding author upon reasonable request.
